# Head-to-Head Comparison of Family History of Colorectal Cancer and a Genetic Risk Score for Colorectal Cancer Risk Stratification

**DOI:** 10.14309/ctg.0000000000000106

**Published:** 2019-11-28

**Authors:** Korbinian Weigl, Li Hsu, Phillip Knebel, Michael Hoffmeister, Maria Timofeeva, Susan Farrington, Malcolm Dunlop, Hermann Brenner

**Affiliations:** 1 Division of Clinical Epidemiology and Aging Research, German Cancer Research Center (DKFZ), Heidelberg, Germany;; 2 German Cancer Consortium (DKTK), German Cancer Research Center (DKFZ), Heidelberg, Germany;; 3 Medical Faculty Heidelberg, University of Heidelberg, Germany;; 4 Public Health Sciences Division, Fred Hutchinson Cancer Research Center, Seattle, Washington, USA;; 5 Department for General, Visceral and Transplantation Surgery, University Heidelberg, Germany;; 6 Colon Cancer Genetics Group, Institute of Genetics and Molecular Medicine, University of Edinburgh and MRC Human Genetics Unit, Western General Hospital Colon Cancer Genetics Group, Edinburgh, Scotland, United Kingdom;; 7 Division of Preventive Oncology, German Cancer Research Center (DKFZ) and National Center of Tumour Diseases (NCT), Heidelberg, Germany.

## Abstract

**OBJECTIVES:**

Family history (FH) is associated with increased risk of colorectal cancer (CRC). We aimed to examine the potential for CRC risk stratification by known common genetic variants beyond FH in a large population-based case-control study from Germany.

**METHODS:**

Four thousand four hundred forty-seven cases and 3,480 controls recruited in 2003–2016 were included for whom comprehensive interview, medical, and genomic data were available. Associations with CRC risk were estimated from multiple logistic regression models for FH and a genetic risk score (GRS) based on 90 previously identified risk variants.

**RESULTS:**

CRC in a first-degree relative was associated with a 1.71-fold (95% confidence interval 1.47–2.00) increase in CRC risk. A higher risk increase (odds ratio 2.06, 95% confidence interval 1.78–2.39) was estimated for the GRS when it was dichotomized at a cutoff yielding the same positivity rate as FH among controls. Furthermore, the GRS provides substantial additional risk stratification in both people with and especially without FH. Equal or even slightly higher risks were observed for participants without FH with a GRS in the upper 20% compared with participants with FH with a GRS below median. The observed patterns were confirmed in a replication study.

**DISCUSSION:**

In contrast to common perception, known genetic variants do not primarily reflect some minor share of the familial excess risk of CRC, but rather reflect a substantial share of risk independent of FH.

## INTRODUCTION

With an estimated toll of 1.8 million new cases and 881.000 deaths in 2018, colorectal cancer (CRC) is the third most common cancer and the second most common cancer cause of death globally (1). It has long been noted that first-degree relatives (FDRs) of patients with CRC have a strongly increased risk of CRC (2), and guidelines for CRC screening consistently recommend to start CRC screening at a younger age in this high-risk population than in the average-risk population (3).

The excess risk among relatives of patients with CRC is generally assumed to be explained to a large extent by shared genetic risk variants (4). A large number of genetic risk variants for CRC have been identified by genomewide association studies (GWASs) (e.g., (5–10)). Although the risk associated with each of the identified common single nucleotide polymorphisms (SNPs) is generally very small, construction of genetic risk scores (GRSs) based on absolute or weighted numbers of risk alleles from multiple SNPs has been shown to be a promising avenue for effective risk stratification in CRC screening (11–13).

Although we were already able to confirm these previous results and additionally show that combination of both family history (FH) and common genetic variants enhances CRC risk stratification (14), the question about the potential of these variants in direct comparison with FH remains unanswered. Similarly, the magnitude to which risk stratification by common genetic variants in both people with and without FH (the only risk stratification criterion commonly used in various CRC guidelines) could be used has not yet been addressed. In this article, we aimed for evaluating the potential for CRC risk stratification by common genetic risk variants identified by GWAS beyond the commonly used risk stratification by FH.

## METHODS

### Study design and study population

Data for the analyses were drawn from the DACHS study (*Darmkrebs: Chancen der Verhütung durch Screening*) which has been described in detail elsewhere (15,16). In brief, DACHS is an ongoing population-based case-control study in the Rhine-Neckar region in southwest Germany. German-speaking patients aged 30 years or older with a first diagnosis of CRC who are capable of taking part in a personal interview of approximately 1 hour are eligible for participation. Physicians inform patients with a first diagnosis of CRC about the study. Approximately 50% of all eligible patients in the study area are recruited. Using frequency matching with respect to sex, age, and county of residence, controls are randomly selected from population registries. Besides excluding persons with a history of CRC among controls, inclusion and exclusion criteria are identical for both cases and controls. This analysis is based on 4,447 cases and 3,480 controls who were recruited from 2003 to 2016 and for whom data from GWAS were available. The participation rate among controls was 51%. The ethics committees of the Medical Faculty at the University of Heidelberg (310/2001) and the Medical Chambers of Baden-Württemberg and Rhineland-Palatinate approved the study. Written informed consent was obtained from all participants.

### Data collection

The study center was informed about cases after they had given written informed consent. Controls were contacted by mail and follow-up telephone calls. Standardized in-person interviews were conducted with both cases (typically during their hospital stay) and controls (at their homes) by trained interviewers. In these interviews, blood or buccal samples were collected, and a broad variety of risk factors and preventive factors for CRC were addressed in great detail. In particular, detailed information about the participants' FH was collected (degree of kinship, number of affected relatives, and age at diagnosis). All cases with primary CRC were histologically confirmed.

### Genotyping

DNA was extracted from blood samples (in 99.1% of participants) or from buccal cells (in 0.9% of participants) using conventional methods. Details about genotyping and imputation of missing genotypes are provided in Table 1 of the Supplementary Digital Content 1 (http://links.lww.com/CTG/A134).

### Identification and selection of SNPs for the GRS

We considered a most recently reported set of 95 SNPs that were identified to be associated with a higher risk of CRC in the world's largest CRC GWAS in populations of European descent (17). Of the reported 95 SNPs, a total of 90 SNPs could be extracted from our data set (see Table 2 of Supplementary Digital Content 1, http://links.lww.com/CTG/A134). The GRS was calculated as the sum of risk alleles (as reported by Huyghe et al. (17)).

### Statistical analysis

We first described the study population according to the distribution of sex, age, FH of CRC, and the GRS. We compared the distribution of the GRS between cases and controls and between participants with and without FH (latter both among cases and among controls). Differences between groups were tested for statistical significance by the Mann-Whitney *U* test.

We then assessed the associations of FH with CRC risk using multiple logistic regression models and adjusting for covariates that are known or suspected to be associated with CRC risk (see Table 2 for description). Associations were quantified by adjusted odds ratios (ORs) and their 95% confidence intervals (CIs).

**Table 1.**
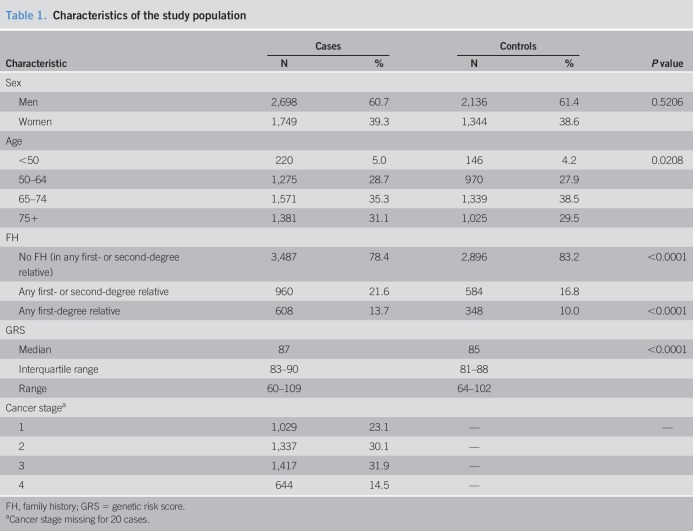
Characteristics of the study population

For comparison reasons, we then performed analogous analyses for the GRS, for which we dichotomized participants according to whether or not their GRS exceeded a defined cutoff (selected in such a way that the proportions of participants in the control group exceeding the cutoff equaled the corresponding proportions meeting the respective FH criteria).

We then examined the joint association of the GRS and FH with the risk of CRC. For this purpose, we divided participants with a positive FH in FDR into 2 groups (equal to or smaller vs greater than median number of risk alleles in controls with FH, n = 86). To allow for meaningful comparisons especially in the high-risk and low-risk group, we then categorized persons without FH into 7 GRS groups with cutoffs at the 10th, 20th, 40th, 60th, 80th, and 90th percentile of the risk alleles among controls without FH. Compared with the reference group of participants without FH and GRS in the bottom decile, we estimated the CRC risk of all other groups. Additional analyses were conducted which examined the association of GRS with CRC risk according to participants' history of colonoscopy.

All statistical analyses were conducted using SAS software, version 9.4 (SAS Institute, Cary, NC) and R statistical software package (18). Two-sided *P* values less than 0.05 were considered statistically significant.

### Replication analyses

Main analyses were replicated using the Study of Colorectal Cancer in Scotland (SOCCS), a population-based case-control study conducted between 1996 and 2006. Details of study recruitment and data collection have been previously described in detail (19). The study included 1,556 incident CRC cases, and 2,201 adult cancer-free controls, identified from the Community Health Index in Scotland. Genotyping was conducted using custom Illumina Infinium arrays and OmniExpressExome BeadChip arrays. Detailed descriptions of genotyping and imputation of common genetic variants are presented in previous reports (20,21). The study was approved by the Multicenter Research Ethics Committee for Scotland (reference number: 01/0/05) and by the Research and Development Office of NHS Lothian (reference number: 2003/W/GEN/05).

## RESULTS

**Figure 1.**
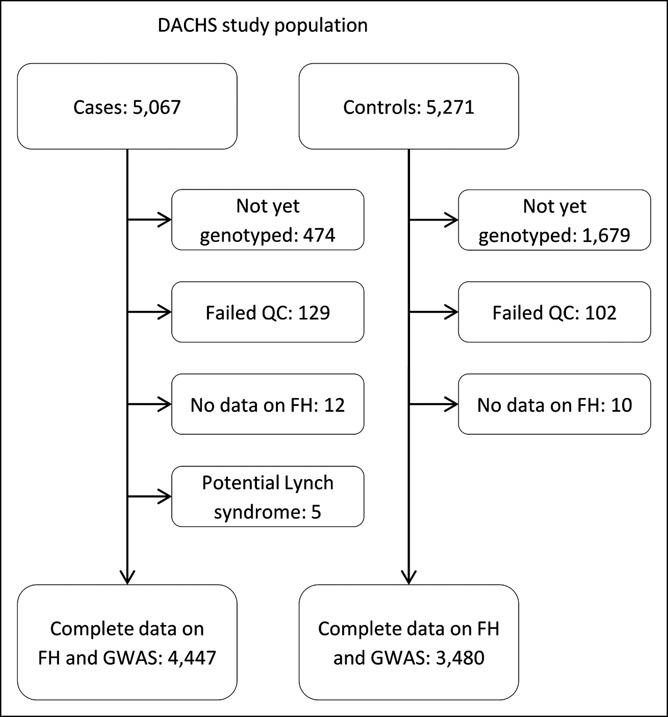
Flow diagram of study participants. FH, family history; GWAS, genomewide association study; QC, quality control.

Of 5,067 cases and 5,271 controls who were recruited in DACHS until 2016, a total of 4,447 cases and 3,480 controls have been genotyped, fulfilled quality control criteria, and had complete data on FH. We excluded 5 cases who fulfilled the Amsterdam criteria and who were potential carriers of the Lynch syndrome (Figure 1).

Table 1 shows some main characteristics of the study population. Approximately 60% of both cases and controls were men. The age distribution was similar among cases and controls (median age: cases 69 years and controls 70 years), as expected as a result of matching. One of 10 controls (10.0%) had an FDR with CRC, and a positive FH was more common among cases than among controls according to all FH definitions. The median GRS was 87 among cases and 85 among controls (*P* < 0.0001) (Table 1, see Figure 1 of Supplementary Digital Content 1, http://links.lww.com/CTG/A134). Substantially smaller but still statistically significant differences were seen between those with and those without an FH of CRC in any FDR, both within controls and within cases (see Figure 1 of Supplementary Digital Content 1, http://links.lww.com/CTG/A134).

**Table 2.**
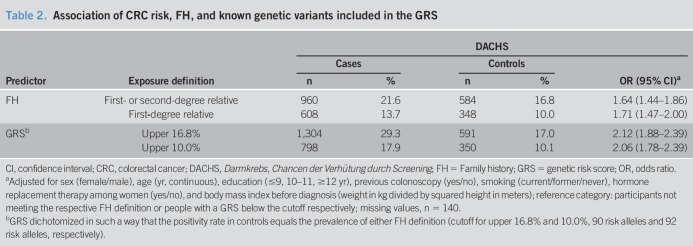
Association of CRC risk, FH, and known genetic variants included in the GRS

Table 2 shows the ORs for the associations between FH and GRS and CRC risk. ORs for FH in DACHS were 1.64 (95% CI 1.44–1.86) for any first- or second-degree relative with CRC and 1.71 (95% CI 1.47–2.00) for having an FDR. Higher ORs were estimated for the GRS when it was dichotomized at levels yielding the same proportions of affected controls as the 2 FH definitions (ORs 2.12, 95% CI 1.88–2.39 and 2.06, 95% CI 1.78–2.39, respectively).

**Figure 2.**
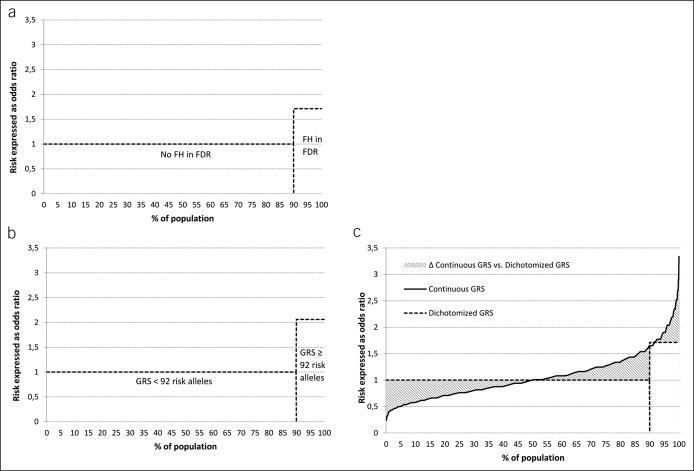
CRC risk associated with FH in FDR and GRS and affected proportions of population. (**a**) CRC risk and affected proportion of FH in FDR. (**b**) CRC risk and affected proportion of dichotomized GRS. (**c**) CRC risk and affected proportion of dichotomized and continuous GRS. CRC, colorectal cancer; FDR, first-degree relative; FH, family history; GRS, genetic risk score; OR, odd ratio.

A comparison of the potential of CRC risk stratification with either information about FH in an FDR or the GRS is shown in Figure 2. Information obtained from the binary FH status yielded increased risk of the small proportion of persons with an affected relative compared to persons without an affected relative (Figure 2a). Dichotomizing the GRS so that the prevalence of exposed participants equals the prevalence of an FH in an FDR resulted in a very similar outcome, with higher odds for the exposed group (Figure 2b). However, much information that can be used for enhanced and refined risk stratification is lost in the dichotomized GRS compared with the continuous GRS (Figure 2c, hatched area).

Table 3 shows the joint association of FH and the GRS with the risk of CRC. Compared with the reference group of persons without FH and the lowest genetic risk (bottom decile of number of risk alleles among controls without FH), people with an FH have a strongly increased risk of CRC (OR 4.65, 95% CI 3.51–6.14 and OR 2.76, 95% CI 2.06–3.69 for people with higher genetic risk and for people with lower genetic risk, respectively). Among those with no FH, the CRC risk steadily increases with increasing GRS up to ORs for people in the top 2 GRS groups of 3.88 (95% CI 3.03–4.96) and 3.16 (95% CI 2.46–4.06), i.e., to ORs comparable with those of the half of people with FH with lower GRS.

**Table 3.**
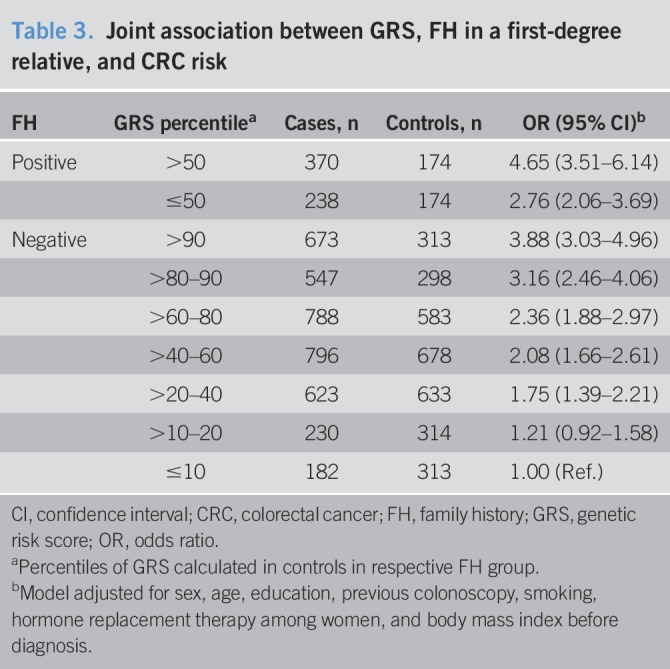
Joint association between GRS, FH in a first-degree relative, and CRC risk

Sensitivity analyses stratifying by cancer stage (see Table 2 of Supplementary Digital Content 1, http://links.lww.com/CTG/A134) showed similar results for each cancer stage, with slightly higher risk estimates for stage 4.

Stratified analyses by age of participant confirmed the overall pattern for all age groups, but revealed an especially pronounced CRC risk increase for younger participants (see Table 4 of Supplementary Digital Content 1, http://links.lww.com/CTG/A134).

Replication analyses of the main results in SOCCS are presented in Tables 6–8 and in the Figures 2 and 3 of the Supplementary Digital Content 1 (http://links.lww.com/CTG/A134). Overall, the population in the SOCCS was younger compared with DACHS participants, and more cases had an FH in an FDR (17.1% vs 13.7% in DACHS, Appendix Table 6, http://links.lww.com/CTG/A134). Because data on second-degree relatives were not available in SOCCS, replication analyses focused on FDR. A higher OR for CRC associated with having an FH in an FDR was obtained in SOCCS compared with DACHS (2.19, 95% CI 1.79–2.70), but dichotomizing the GRS to generate the same proportion of exposure yielded a comparable OR compared with FH (2.07, 95% CI 1.69–2.53). Overall, the joint association of GRS and FH with CRC risk in SOCCS yielded similar results as analyses in DACHS. Figure 2 of the Supplementary Digital Content (http://links.lww.com/CTG/A134) confirms the patterns observed in DACHS, with major differences in the GRS distribution between cases and controls, but not at all or not as distinct between persons with and without FH in either cases or controls, respectively. The replication of the enhanced risk stratification by a continuous GRS compared with binary variables such as FH is depicted in Figure 3 of the Supplementary Digital Content 1 (http://links.lww.com/CTG/A134).

## DISCUSSION

In this large case-control study, we aimed for an evaluation of the associations of FH and a GRS based on common genetic variants with the risk of CRC. Although most FH definitions are restricted to 2 groups (those with and those without FH), a GRS provides information over the complete spectrum of its range. Taking this information into account, the GRS adds valuable information with respect to risk stratification especially in persons without FH, some proportion of whom have as high or even higher risk than those with a positive FH. We could replicate our findings in an independent cohort.

Our analyses showed that 20% of persons without FH (10% in the replication cohort, respectively) have at least a comparable CRC risk because of their increased genetic risk compared with 50% of persons with an FH in an FDR (i.e., those with FH, but GRS below the median). Considering that persons without FH are constituting 90% of the population, 20% of this group is a larger share of the total population (approximately 18%) than half the population with an FH (approximately 5%), for whom CRC risk is deemed to be increased in a way that earlier screening initiation is recommended. Arguably, comparable CRC risk should yield comparable CRC screening guidelines. Potential implications for risk stratification could be as follows: while people with an FH of CRC should be advised to start screening earlier as commonly recommended, people without FH might be offered determination of a GRS for risk stratification, in that those above the 80th percentile might be advised to start screening earlier, similar to those with an FH, and those with lower GRS might be advised to follow recommendations for the average risk population. It remains to be clarified how risk stratification with a GRS could be practically implemented in existing health care models, especially given the sensitive nature of genetic testing. One possibility could be to merely communicate a quantitative measure (e.g., solely the sum of risk alleles) and the associated CRC risk, which may be translated in a personalized recommendation for the starting age of CRC screening, to avoid unnecessary concern about the individuals' genetic condition. Approaches like these should however be indeed evaluated, especially considering that the positive FH criterion could become less important in future risk stratification approaches if persons meeting screening recommendations actually participate in CRC screening (22). Fewer people would then exhibit an FH of CRC leaving behind many persons without FH for whom no risk-adapted screening recommendations are yet established.

The observed associations also show that it is misleading to think of genetic risk only in the context of a positive FH. Instead, as illustrated by our results, the GRS not just explains a small proportion of the excess risk associated with FH. Rather, it is responsible for a large proportion of the high end CRC risk in the population in general. Moreover, whereas FH only conveys risk information at the upper end for a small high-risk proportion, the GRS provides information beyond dichotomization and offers risk stratification potential over the full range of its distribution in the population, most importantly also in persons without FH. The GRS can hence not only be used to identify people at highest risk in whom initiating screening at younger ages or in shorter intervals might be warranted but also it provides the opportunity to help identifying low-risk individuals for whom CRC screening could start at a later age or be less intensive.

Our study has a number of specific strengths and limitations. Strengths include the large sample size and unselected population-based multicenter recruitment of cases and controls in a defined large area. Furthermore, in contrast to most other studies, no upper age limit was used. Because many CRC cases occur at relatively old ages (mean ages at diagnosis are 75 years for women and 69 years for men), our results should provide a more comprehensive estimation of ORs than studies from selective academic centers or population subgroups. The analyses were furthermore replicated in an independent cohort from Scotland.

On the other hand, we cannot rule out some selection bias in DACHS by recruitment of approximately 50% of eligible cases and controls only. Main reasons for nonrecruitment of cases were overload of their treating physicians (through whom recruitment had to be realized) or patients being too sick to be able to participate, and the latter was also a major reason of nonparticipation of controls, especially at older ages. There seems to be no obvious reason, however, to assume that these factors were related to FH or the GRS, the main variables of interest in our analysis. Information on FH was self-reported and may be subject to lack of awareness and imperfect recall and reporting. In particular, cases may be more aware of a positive FH which might have led to some overestimation of the associations of FH with CRC risk. However, previous studies have shown self-reported CRC in an FDR to be rather accurate (23,24), with little variation between cases and controls (23,25), suggesting that the potential bias is likely to be limited (23–25). Although the observed associations of SNPs often reflect indirect effects, which diminish the use of SNPs and the resulting GRS for causal inferences, it does not necessarily limit the use of GRS for risk prediction and risk stratification. We used unweighted GRS for this analysis (i.e., number of risk alleles), but previous research suggests that weighted GRS (i.e., weighting SNPs with respective log[OR]) yields comparable results (12,26). Although our study sample was large, sample size issues limited more refined GRS groups, especially in the FH positive population. Our results pertain to a study population of almost exclusively white origin. Although GRS for other ethnicities is likely to differ with respect to the variants included (e.g., (27)), it seems unlikely that the observed patterns of complementary risk information by FH and GRS should be substantially different in other ethnic groups.

The presented results confirm previous analyses conducted in a subset of this study population (14) in that CRC risk stratification might strongly benefit from including information about GRS together with FH. Given the larger sample size, we were able to examine in more detail the direct comparison between GRS and FH, which was not possible in the previous analysis.

In conclusion, our study demonstrates the complementary character of an FH of CRC and meanwhile identified common genetic variants on CRC risk. These genetic variants do not primarily reflect some minor share of the familial excess risk, but rather reflect most relevant risk information independent of the risk that has previously become manifest in the family. GRS might therefore be useful supplements to existing CRC risk scores or risk stratification criteria far beyond FH which is already included in most of them.

## CONFLICTS OF INTEREST

**Guarantor of the article:** Korbinian Weigl, PhD.

**Specific author contributions:** K.W.: statistical analysis and interpretation of data and drafting of the manuscript. L.H.: critical revision of the manuscript for important intellectual content and supervision of genotyping within the GECCO consortium. P.K.: critical revision of the manuscript for important intellectual content. M.H.: DACHS study coordination and supervision, critical revision of the manuscript for important intellectual content, and obtained funding. M.T.: statistical analysis and critical revision of the manuscript for important intellectual content. S.F. and M.D.: concept and design of the work in Scotland, supervision and funding, and critical revision of the manuscript for important intellectual content. H.B.: DACHS study design and supervision, drafting of the manuscript, critical revision of the manuscript for important intellectual content, and obtained funding. All authors had full access to all data (including statistical reports and tables) in the study and can take responsibility for the integrity of the data and the accuracy of the data analysis.

**Financial support:** This work was supported by grants from the German Research Council (BR 1704/6-1, BR 1704/6-3, BR 1704/6-4, BR 1704/6-6, CH 117/1-1, HO 5117/2-1, HE 5998/2-1, KL 2354/3-1, RO 2270/8-1, BR 1704/17-1), the German Federal Ministry of Education and Research (01KH0404, 01ER0814, 01GL1712), the Interdisciplinary Research Program of the National Center for Tumor Diseases (NCT), Germany, and the National Cancer Institute, National Institutes of Health, and US Department of Health and Human Services (NIH R01 CA195789, U01 CA137088, R01 CA059045, and U01 CA185094). The work in Scotland was funded by a Program Grant from Cancer Research UK (C348/A18927), Scottish Colorectal Cancer Study (SOCCS).

**Potential competing interests:** All authors have completed the ICMJE uniform disclosure form at www.icmje.org/coi_disclosure.pfd and declare no support from any organization for the submitted work; HB has received research grants from the German Federal Ministry of Education and Research and from the German Research Council (Deutsche Forschungsgemeinschaft, DFG) during the conduct of the study; no financial relationships with any organizations that might have an interest in the submitted work in the previous 3 years; and no other relationships or activities that could appear to have influenced the submitted work.

**Study Highlights**

**WHAT IS KNOWN**

✓ Having an FH of CRC increases the risk of CRC and is therefore used as a risk stratification criterion in pertinent guidelines.
✓ Many common genetic variants have been found to increase the risk of CRC.

**WHAT IS NEW HERE**

✓ Known genetic variants do not primarily reflect some minor share of the familial excess risk of CRC, but rather reflect a substantial share of risk independent of FH.
✓ Using both information on common genetic variants and a self-reported FH substantially enhances risk stratification, especially for individuals without FH, for whom no risk-adapted prevention strategies are currently available.

**TRANSLATIONAL IMPACT**

✓ Genetic risk scores could help to identify high risk individuals for whom no risk-adapted screening strategies are yet developed but who carry similar risks to people for whom such strategies are commonly used.

## Supplementary Material

SUPPLEMENTARY MATERIAL
