# Supplementary material for: Head-to-Head Comparison of Family History of Colorectal Cancer and a Genetic Risk Score for Colorectal Cancer Risk Stratification
Source: Clin Transl Gastroenterol. 2019 Nov 28;10(12):e00106. doi: 10.14309/ctg.0000000000000106 (PMC6970558; doi:10.14309/ctg.0000000000000106)
Supplement: SUPPLEMENTARY MATERIAL [file ct9-10-e00106-s001.docx]

**Supplementary Material “FH, common genetic variants and the risk of colorectal cancer”**

**Supplementary Table 1.** Information about genotyping and imputation within DACHS

**Supplementary Table 2.** Information about SNPs used to generate the genetic risk score (GRS)

**Supplementary Table 3.** Joint association between genetic risk score (GRS), family history (FH) and colorectal cancer risk (stratified by cancer stage)

**Supplementary Table 4.** Joint association between genetic risk score (GRS), family history (FH) and colorectal cancer risk (stratified by age of individual)

**Supplementary Table 5.** Characteristics of the replication study (Study of Colorectal Cancer in Scotland, SOCCS)

**Supplementary Table 6.** Association of colorectal cancer risk, family history and known genetic variants included in the genetic risk score (GRS) in SOCCS

**Supplementary Table 7.** Joint association between genetic risk score (GRS), family history (FH) and colorectal cancer risk in SOCCS

**Supplementary Table 8** Joint association between genetic risk score (GRS), family history (FH) and colorectal cancer risk (stratified by cancer stage) in SOCCS

**Supplementary Figure 1.** Scatter plot and box plot showing the distribution of GRS between different study groups in DACHS

**Supplementary Figure 2.** Scatter plot and box plot showing the distribution of GRS between different study groups in SOCCS

**Supplementary Figure 3.** CRC risk associated with family history (FH) in first-degree relatives (FDR) and genetic risk score (GRS) and affected proportions of population in SOCCS

**Supplementary Table 1.** Information about genotyping and imputation within DACHS

| Genotyping platform | Number of cases | Number of controls | Recruitment period | Imputation |
| --- | --- | --- | --- | --- |
|  |  |  |  |  |
| Illumina HumanCytoSNP | 1,694 | 1,700 | 2003-2008 | Cosmopolitan panel of reference haplotypes from Phase 1 of the 1,000 Genome Project |
| Illumina HumanOmniExpress | 668 | 498 | 2007-2010 |  |
| Illumina HumanOmniExpress | 1,191 | 627 | 2010-2015 | Haplotype Reference Consortium (Version r1.1.2016) |
| Illumina Infinium OncoArray | 894 | 655 | 2003-2016 |  |

Triallelic SNPs and those not assigned an rs-number were excluded, as were genotyped SNPs when they had a low call rate (<98%), lack of Hardy-Weinberg equilibrium in controls (p<1x10^-4^) or low minor allele frequency (<0.1%). See (1) and (2) for more information about genotyping and imputation. Please note that data from Illumina HumanCytoSNP and the Illumina HumanOmniExpress with recruitment period from 2007-2010 have been used for previous analyses within the DACHS study (3).

**Supplementary Table 2.** Information about SNPs used to generate the genetic risk score (GRS), adapted from Huyghe et al. (4)

| **Locus** | **rsID lead variant** | **Variant** | **Chr.** | **Position (Build 37)** | **Risk allele** | **Other allele** | **Available in DACHS** |
| --- | --- | --- | --- | --- | --- | --- | --- |
| 1p36.12 | rs72647484 | 1:22587728_T/C | 1 | 22.587.728 | T | C | Yes |
| 1p34.3 | rs4360494 | 1:38455891_G/C | 1 | 38.455.891 | G | C | Yes |
| 1p32.3 | rs12144319 | 1:55246035_T/C | 1 | 55.246.035 | C | T | Yes |
| 1q25.3 | rs6678517 | 1:183002639_A/G | 1 | 183.002.639 | A | G | Yes |
| 1q41 | rs17011141 | 1:222112634_A/G | 1 | 222.112.634 | G | A | Yes |
| 2q24.2 | rs448513 | 2:159964552_T/C | 2 | 159.964.552 | C | T | Yes |
| 2q33.1 | rs11884596 | 2:199612407_T/C | 2 | 199.612.407 | C | T | Yes |
| 2q33.1 | rs983402 | 2:199781586_T/C | 2 | 199.781.586 | T | C | Yes |
| 2q35 | rs3731861 | 2:219191256_T/C | 2 | 219.191.256 | T | C | Yes |
| 3p22.1 | rs35470271 | 3:40915239_A/G | 3 | 40.915.239 | G | A | Yes |
| 3p14.1 | rs6781752 | 3:66365163_G/A | 3 | 66.365.163 | A | G | Yes |
| 3q13.2 | rs72942485 | 3:112999560_G/A | 3 | 112.999.560 | G | A | Yes |
| 3q22.2 | rs10049390 | 3:133701119_G/A | 3 | 133.701.119 | A | G | Yes |
| 3q26.2 | rs9876206 | 3:169517436_C/T | 3 | 169.517.436 | C | T | Yes |
| 4q22.2 | rs13149359 | 4:94938618_C/A | 4 | 94.938.618 | A | C | Yes |
| 4q24 | rs1391441 | 4:106128760_G/A | 4 | 106.128.760 | A | G | Yes |
| 4q31.21 | rs11727676 | 4:145659064_T/C | 4 | 145.659.064 | C | T | Yes |
| 5p15.33 | rs78368589 | 5:1240204_C/T | 5 | 1.240.204 | T | C | Yes |
| 5p15.33 | rs2735940 | 5:1296486_A/G | 5 | 1.296.486 | G | A | Yes |
| 5p13.1 | rs7708610 | 5:40102443_G/A | 5 | 40.102.443 | A | G | Yes |
| 5p13.1 | rs12514517 | 5:40280076_G/A | 5 | 40.280.076 | A | G | Yes |
| 5q21.1 | rs145364999 | 5:98206082_T/A | 5 | 98.206.082 | T | A | Yes |
| 5q22.2 | rs755229494 | 5:112097351_A/G | 5 | 112.097.351 | G | A | No |
| 5q31.1 | rs4976270 | 5:134467220_C/T | 5 | 134.467.220 | C | T | Yes |
| 6p21.33 | rs2516420 | 6:31449620_C/T | 6 | 31.449.620 | C | T | Yes |
| 6p21.32 | rs9271695 | 6:32593080_A/G | 6 | 32.593.080 | G | A | No |
| 6p21.31 | rs16878812 | 6:35569562_A/G | 6 | 35.569.562 | A | G | Yes |
| 6p21.2 | rs9470361 | 6:36623379_G/A | 6 | 36.623.379 | A | G | Yes |
| 6p21.1 | rs62396735 | 6:41702582_C/T | 6 | 41.702.582 | C | T | Yes |
| 6p12.1 | rs62404966 | 6:55712124_C/T | 6 | 55.712.124 | C | T | Yes |
| 7p13 | rs12672022 | 7:45136423_T/C | 7 | 45.136.423 | T | C | Proxy in perfect linkage disequilibrium: rs145013958 |
| 8q23.3 | rs16892766 | 8:117630683_A/C | 8 | 117.630.683 | C | A | Yes |
| 8q23.3 | rs6469654 | 8:117632965_G/C | 8 | 117.632.965 | G | C | Yes |
| 8q24.11 | rs117079142 | 8:117790914_C/A | 8 | 117.790.914 | A | C | Yes |
| 8q24.21 | rs6983267 | 8:128413305_G/T | 8 | 128.413.305 | G | T | Yes |
| 8q24.21 | rs4313119 | 8:128571855_G/T | 8 | 128.571.855 | G | T | Yes |
| 9p21.3 | rs1537372 | 9:22103183_G/T | 9 | 22.103.183 | G | T | Yes |
| 9q22.33 | rs34405347 | 9:101679752_T/G | 9 | 101.679.752 | T | G | Yes |
| 9q31.3 | rs10980628 | 9:113671403_T/C | 9 | 113.671.403 | C | T | Yes |
| 10p14 | rs11255841 | 10:8739580_T/A | 10 | 8.739.580 | T | A | Yes |
| 10q11.23 | rs10821907 | 10:52648454_C/T | 10 | 52.648.454 | C | T | Yes |
| 10q22.3 | rs704017 | 10:80819132_A/G | 10 | 80.819.132 | G | A | Yes |
| 10q24.2 | rs11190164 | 10:101351704_A/G | 10 | 101.351.704 | G | A | Yes |
| 10q25.2 | rs12246635 | 10:114288619_T/C | 10 | 114.288.619 | C | T | Yes |
| 10q25.2 | rs11196170 | 10:114722621_G/A | 10 | 114.722.621 | A | G | Yes |
| 11q12.2 | rs174533 | 11:61549025_G/A | 11 | 61.549.025 | G | A | Yes |
| 11q13.4 | rs7121958 | 11:74280012_T/G | 11 | 74.280.012 | G | T | Yes |
| 11q13.4 | rs61389091 | 11:74427921_C/T | 11 | 74.427.921 | C | T | Yes |
| 11q22.1 | rs2186607 | 11:101656397_T/A | 11 | 101.656.397 | T | A | Yes |
| 11q23.1 | rs3087967 | 11:111156836_T/C | 11 | 111.156.836 | T | C | Yes |
| 12p13.32 | rs35808169 | 12:4368607_T/C | 12 | 4.368.607 | C | T | Yes |
| 12p13.32 | rs3217810 | 12:4388271_C/T | 12 | 4.388.271 | T | C | Yes |
| 12p13.32 | rs3217874 | 12:4400808_C/T | 12 | 4.400.808 | T | C | Yes |
| 12p13.31 | rs2250430 | 12:6421174_A/T | 12 | 6.421.174 | T | A | Yes |
| 12p13.2 | rs2710310 | 12:12035649_C/T | 12 | 12.035.649 | C | T | Yes |
| 12q12 | rs11610543 | 12:43134191_A/G | 12 | 43.134.191 | G | A | Yes |
| 12q13.12 | rs12372718 | 12:51171090_A/G | 12 | 51.171.090 | G | A | Yes |
| 12q13.3 | rs4759277 | 12:57533690_C/A | 12 | 57.533.690 | A | C | Yes |
| 12q24.12 | rs597808 | 12:111973358_A/G | 12 | 111.973.358 | G | A | Yes |
| 12q24.21 | rs7300312 | 12:115890922_T/C | 12 | 115.890.922 | C | T | Yes |
| 13q13.2 | rs377429877 | 13:34092164_C/T | 13 | 34.092.164 | C | T | No |
| 13q13.3 | rs7333607 | 13:37462010_A/G | 13 | 37.462.010 | G | A | Yes |
| 13q22.1 | rs78341008 | 13:73791554_T/C | 13 | 73.791.554 | C | T | Yes |
| 13q34 | rs8000189 | 13:111075881_C/T | 13 | 111.075.881 | T | C | Yes |
| 14q22.2 | rs35107139 | 14:54419106_A/C | 14 | 54.419.106 | C | A | Yes |
| 14q22.2 | rs4901473 | 14:54445157_G/A | 14 | 54.445.157 | G | A | Yes |
| 14q23.1 | rs17094983 | 14:59189361_G/A | 14 | 59.189.361 | G | A | Yes |
| 15q13.3 | rs12708491 | 15:32992836_G/A | 15 | 32.992.836 | G | A | Yes |
| 15q13.3 | rs2293581 | 15:33010736_G/A | 15 | 33.010.736 | A | G | Yes |
| 15q13.3 | rs17816465 | 15:33156386_G/A | 15 | 33.156.386 | A | G | Yes |
| 15q22.33 | rs56324967 | 15:67402824_T/C | 15 | 67.402.824 | C | T | Yes |
| 16q22.1 | rs9924886 | 16:68743939_A/C | 16 | 68.743.939 | A | C | Yes |
| 16q23.2 | rs9930005 | 16:80043258_C/A | 16 | 80.043.258 | C | A | Yes |
| 16q24.1 | rs12149163 | 16:86339315_T/C | 16 | 86.339.315 | T | C | Yes |
| 16q24.1 | rs62042090 | 16:86703949_C/T | 16 | 86.703.949 | T | C | Yes |
| 17p13.3 | rs4968127 | 17:809643_G/A | 17 | 809.643 | G | A | Yes |
| 17p12 | rs1078643 | 17:10707241_G/A | 17 | 10.707.241 | A | G | Yes |
| 17q24.3 | rs983318 | 17:70413253_G/A | 17 | 70.413.253 | A | G | Yes |
| 17q25.3 | rs75954926 | 17:81061048_A/G | 17 | 81.061.048 | G | A | No |
| 18q21.1 | rs11874392 | 18:46453156_A/T | 18 | 46.453.156 | A | T | Yes |
| 19p13.11 | rs34797592 | 19:16417198_C/T | 19 | 16.417.198 | T | C | Yes |
| 19q13.11 | rs28840750 | 19:33519927_T/G | 19 | 33.519.927 | T | G | Yes |
| 19q13.2 | rs1963413 | 19:41871573_G/A | 19 | 41.871.573 | A | G | Yes |
| 19q13.43 | rs73068325 | 19:59079096_C/T | 19 | 59.079.096 | T | C | Yes |
| 20p12.3 | rs189583 | 20:6376457_G/C | 20 | 6.376.457 | G | C | Yes |
| 20p12.3 | rs994308 | 20:6603622_C/T | 20 | 6.603.622 | C | T | Yes |
| 20p12.3 | rs4813802 | 20:6699595_T/G | 20 | 6.699.595 | G | T | Yes |
| 20p12.3 | rs28488 | 20:6762221_C/T | 20 | 6.762.221 | T | C | Yes |
| 20p12.3 | rs11087784 | 20:7740976_A/G | 20 | 7.740.976 | G | A | Yes |
| 20q11.22 | rs6058093 | 20:33213196_A/C | 20 | 33.213.196 | C | A | Yes |
| 20q13.12 | rs6031311 | 20:42666475_C/T | 20 | 42.666.475 | T | C | Yes |
| 20q13.13 | rs6066825 | 20:47340117_A/G | 20 | 47.340.117 | A | G | Yes |
| 20q13.13 | rs6063514 | 20:49055318_C/T | 20 | 49.055.318 | C | T | Yes |
| 20q13.33 | rs1741640 | 20:60932414_T/C | 20 | 60.932.414 | C | T | No |
| 20q13.33 | rs2738783 | 20:62308612_T/G | 20 | 62.308.612 | T | G | Yes |

**Supplementary Table 3.** Joint association between genetic risk score (GRS), family history (FH) and colorectal cancer risk (stratified by cancer stage)

| CRC stage | FH | GRS percentile^1^ | Cases, n | Controls, n | OR (95% CI)^2^ |
| --- | --- | --- | --- | --- | --- |
|  | | | | | |
| 1 | Positive | >50 | 96 | 193 | 5.63 (3.59-8.82) |
|  |  | ≤50 | 49 | 155 | 3.28 (2.00-5.39) |
|  |  |  |  |  |  |
|  | Negative | >90 | 117 | 270 | 4.14 (2.69-6.37) |
|  |  | >80-90 | 143 | 337 | 3.92 (2.58-5.96) |
|  |  | >60-80 | 176 | 587 | 2.89 (1.92-4.33) |
|  |  | >40-60 | 167 | 660 | 2.45 (1.63-3.67) |
|  |  | >20-40 | 111 | 597 | 1.81 (1.19-2.76) |
|  |  | >10-20 | 60 | 361 | 1.55 (0.98-2.45) |
|  |  | ≤10 | 34 | 320 | **1.00 (Ref.)** |
|  | | | | | |
| 2 | Positive | >50 | 129 | 193 | 5.05 (3.41-7.48) |
|  |  | ≤50 | 58 | 155 | 2.38 (1.52-3.71) |
|  |  |  |  |  |  |
|  | Negative | >90 | 166 | 270 | 3.61 (2.50-5.22) |
|  |  | >80-90 | 161 | 337 | 2.81 (1.95-4.05) |
|  |  | >60-80 | 234 | 587 | 2.29 (1.62-3.23) |
|  |  | >40-60 | 225 | 660 | 1.97 (1.40-2.79) |
|  |  | >20-40 | 186 | 597 | 1.82 (1.28-2.58) |
|  |  | >10-20 | 88 | 361 | 1.34 (0.91-1.99) |
|  |  | ≤10 | 55 | 320 | **1.00 (Ref.)** |
|  | | | | | |
| 3 | Positive | >50 | 121 | 193 | 4.77 (3.23-7.05) |
|  |  | ≤50 | 58 | 155 | 2.34 (1.50-3.65) |
|  |  |  |  |  |  |
|  | Negative | >90 | 190 | 270 | 4.25 (2.96-6.10) |
|  |  | >80-90 | 209 | 337 | 3.63 (2.55-5.16) |
|  |  | >60-80 | 260 | 587 | 2.58 (1.84-3.62) |
|  |  | >40-60 | 235 | 660 | 2.18 (1.55-3.05) |
|  |  | >20-40 | 178 | 597 | 1.85 (1.30-2.61) |
|  |  | >10-20 | 84 | 361 | 1.31 (0.89-1.93) |
|  |  | ≤10 | 57 | 320 | **1.00 (Ref.)** |
|  | | | | | |
| 4 | Positive | >50 | 51 | 193 | 4.59 (2.70-7.80) |
|  |  | ≤50 | 27 | 155 | 2.25 (1.24-4.08) |
|  |  |  |  |  |  |
|  | Negative | >90 | 95 | 270 | 4.21 (2.61-6.80) |
|  |  | >80-90 | 87 | 337 | 3.01 (1.87-4.84) |
|  |  | >60-80 | 110 | 587 | 2.25 (1.42-3.56) |
|  |  | >40-60 | 131 | 660 | 2.30 (1.46-3.62) |
|  |  | >20-40 | 68 | 597 | 1.40 (0.87-2.27) |
|  |  | >10-20 | 48 | 361 | 1.44 (0.86-2.41) |
|  |  | ≤10 | 27 | 320 | **1.00 (Ref.)** |

Abbreviations: CI, confidence interval; FH, family history; GRS, genetic risk score; OR, odds ratio; Ref., reference
^1^ Percentiles of GRS calculated in controls in overall sample
^2^ Model adjusted for sex, age, education, previous colonoscopy, smoking, hormone replacement therapy among women, and body mass index before diagnosis

**Supplementary Table 4.** Joint association between genetic risk score (GRS), family history (FH) and colorectal cancer risk (stratified by age of individual)

| Age of participant | FH | GRS percentile^1^ | Cases, n | Controls, n | OR (95% CI)^2^ |
| --- | --- | --- | --- | --- | --- |
|  | | | | | |
| <50 years | Positive | >50 | 20 | 5 | 12.25 (3.22-46.64) |
|  |  | ≤50 | 6 | 4 | 4.45 (0.90-21.96) |
|  |  |  |  |  |  |
|  | Negative | >90 | 36 | 7 | 14.40 (4.33-47.89) |
|  |  | >80-90 | 34 | 9 | 10.28 (3.24-32.64) |
|  |  | >60-80 | 52 | 24 | 6.44 (2.35-17.67) |
|  |  | >40-60 | 28 | 32 | 2.60 (0.94-7.22) |
|  |  | >20-40 | 24 | 21 | 2.88 (0.99-8.37) |
|  |  | >10-20 | 13 | 22 | 1.57 (0.50-4.89) |
|  |  | ≤10 | 7 | 22 | **1.00 (Ref.)** |
|  | | | | | |
| 50-64 | Positive | >50 | 118 | 52 | 6.42 (3.70-11.15) |
|  |  | ≤50 | 66 | 41 | 3.98 (2.20-7.22) |
|  |  |  |  |  |  |
|  | Negative | >90 | 177 | 76 | 4.87 (2.94-8.06) |
|  |  | >80-90 | 190 | 109 | 3.71 (2.29-6.02) |
|  |  | >60-80 | 213 | 169 | 2.50 (1.57-3.98) |
|  |  | >40-60 | 219 | 180 | 2.48 (1.56-3.95) |
|  |  | >20-40 | 171 | 158 | 2.22 (1.38-3.56) |
|  |  | >10-20 | 82 | 103 | 1.66 (0.99-2.78) |
|  |  | ≤10 | 39 | 82 | **1.00 (Ref.)** |
|  | | | | | |
| 65-74 | Positive | >50 | 135 | 68 | 4.58 (2.90-7.24) |
|  |  | ≤50 | 73 | 57 | 2.38 (1.44-3.93) |
|  |  |  |  |  |  |
|  | Negative | >90 | 216 | 108 | 3.57 (2.37-5.39) |
|  |  | >80-90 | 229 | 123 | 3.49 (2.33-5.23) |
|  |  | >60-80 | 276 | 234 | 2.27 (1.56-3.31) |
|  |  | >40-60 | 293 | 256 | 2.25 (1.55-3.27) |
|  |  | >20-40 | 186 | 249 | 1.44 (0.98-2.11) |
|  |  | >10-20 | 92 | 127 | 1.27 (0.82-1.97) |
|  |  | ≤10 | 71 | 117 | **1.00 (Ref.)** |
|  | | | | | |
| 75+ | Positive | >50 | 135 | 68 | 3.52 (2.20-5.62) |
|  |  | ≤50 | 55 | 53 | 1.75 (1.02-3.00) |
|  |  |  |  |  |  |
|  | Negative | >90 | 161 | 79 | 3.04 (1.93-4.77) |
|  |  | >80-90 | 167 | 96 | 2.54 (1.63-3.95) |
|  |  | >60-80 | 257 | 160 | 2.25 (1.50-3.36) |
|  |  | >40-60 | 248 | 192 | 1.84 (1.24-2.75) |
|  |  | >20-40 | 190 | 169 | 1.66 (1.10-2.50) |
|  |  | >10-20 | 103 | 109 | 1.29 (0.82-2.02) |
|  |  | ≤10 | 65 | 99 | **1.00 (Ref.)** |

Abbreviations: CI, confidence interval; FH, family history; GRS, genetic risk score; OR, odds ratio; Ref., reference
^1^ Percentiles of GRS calculated in controls in overall sample
^2^ Model adjusted for sex, age, education, previous colonoscopy, smoking, hormone replacement therapy among women, and body mass index before diagnosis

**Supplementary Table 5.** Characteristics of the replication study (Study of Colorectal Cancer in Scotland, SOCCS)

| Characteristic | | Cases | | Controls | | p-value |
| --- | --- | --- | --- | --- | --- | --- |
|  |  | N | % | N | % |  |
|  |  |  |  |  |  |  |
| Sex | Men | 890 | 57.2 | 1274 | 57.9 | 0.7003 |
|  | Women | 666 | 42.8 | 927 | 42.1 |  |
|  |  |  |  |  |  |  |
| Age | <50 | 212 | 13.6 | 208 | 9.5 | <.0001 |
|  | 50-64 | 669 | 43.0 | 811 | 36.8 |  |
|  | 65-74 | 526 | 33.8 | 880 | 40.0 |  |
|  | 75+ | 149 | 9.6 | 302 | 13.7 |  |
|  |  |  |  |  |  |  |
| Family history | No family history | 1290 | 82.9 | 1997 | 90.8 | <.0001 |
|  | Any 1^st^ degree relative | 266 | 17.1 | 202 | 9.2 |  |
|  |  |  |  |  |  |  |
| Genetic risk score | Median | 90 |  | 87 |  | <.0001 |
|  | Interquartile range | 82-98 |  | 79-95 |  |  |
|  | Range | 67-111 |  | 69-108 |  |  |
|  |  |  |  |  |  |  |
| Cancer stage^1^ | 1 | 302 | 19.8 |  |  |  |
|  | 2 | 520 | 34.2 |  |  |  |
|  | 3 | 493 | 32.4 |  |  |  |
|  | 4 | 207 | 13.6 |  |  |  |
| ^1^ Cancer stage for 34 cases missing | | | | | | |

**Supplementary Table 6.** Association of colorectal cancer risk, family history and known genetic variants included in the genetic risk score (GRS) in SOCCS

| Predictor | Exposure definition | Cases | | Controls | | OR (95% CI)^1^ |
| --- | --- | --- | --- | --- | --- | --- |
|  |  | n | % | n | % |  |
|  |  |  |  |  |  |  |
| Family history | 1^st^ degree relatives | 266 | 17.1 | 202 | 9.2 | 2.19 (1.79-2.70) |
|  |  |  |  |  |  |  |
| Genetic risk score^2^ | Upper 9.7% | 307 | 19.7 | 198 | 9.0 | 2.49 (2.04-3.06) |
|  | Upper 17.0% | 505 | 32.4 | 369 | 16.8 | 2.34 (1.99-2.75) |
|  |  |  |  |  |  |  |

CI, confidence interval; OR, odds ratio

^1^ Adjusted for sex, age, education, previous colonoscopy, smoking, hormone replacement therapy among women, body mass index before diagnosis; reference category: participants not meeting the family history definition or people with a genetic risk score below the cutoff as defined in the DACHS study, respectively
^2^ Genetic risk score dichotomized in such a way that the positivity rate in controls equals the prevalence of either family history definition (cut-off for upper 16.8% and 9.2%, 94 risk alleles and 97 risk alleles, respectively)

**Supplementary Table 7.** Joint association between genetic risk score (GRS), family history (FH) and colorectal cancer risk in SOCCS

| FH | GRS percentile^1^ | Cases, n | Controls, n | OR (95% CI)^2^ |
| --- | --- | --- | --- | --- |
|  |  |  |  |  |
| Positive | >50 | 179 | 112 | 5.90 (4.01-8.78) |
|  | ≤50 | 87 | 90 | 3.63 (2.37-5.61) |
|  |  |  |  |  |
| Negative | >90 | 288 | 213 | 4.66 (3.29-6.68) |
|  | >80-90 | 176 | 166 | 3.47 (2.4-5.08) |
|  | >60-80 | 273 | 420 | 2.20 (1.57-3.12) |
|  | >40-60 | 210 | 374 | 1.97 (1.4-2.81) |
|  | >20-40 | 223 | 422 | 1.87 (1.33-2.66) |
|  | >10-20 | 62 | 197 | 1.14 (0.75-1.74) |
|  | ≤10 | 58 | 205 | **1.00 (Ref.)** |

Abbreviations: CI, confidence interval; FH, family history; GRS, genetic risk score; OR, odds ratio; Ref., reference
^1^ Percentiles of GRS calculated in controls in overall sample
^2^ Model adjusted for sex, age, education, previous colonoscopy, smoking, hormone replacement therapy among women, and body mass index before diagnosis

**Supplementary Table 8.** Joint association between genetic risk score (GRS), family history (FH) and colorectal cancer risk (stratified by cancer stage) in SOCCS

| CRC stage | FH | GRS percentile^1^ | Cases, n | Controls, n | OR (95% CI)^2^ |
| --- | --- | --- | --- | --- | --- |
|  | | | | | |
| 1 | Positive | >50 | 37 | 112 | 6.48 (3.23-13.98) |
|  |  | ≤50 | 12 | 90 | 2.43 (1-5.93) |
|  |  |  |  |  |  |
|  | Negative | >90 | 57 | 213 | 4.81 (2.51-10.03) |
|  |  | >80-90 | 33 | 166 | 3.35 (1.66-7.22) |
|  |  | >60-80 | 54 | 420 | 2.38 (1.25-4.92) |
|  |  | >40-60 | 39 | 374 | 1.94 (1-4.09) |
|  |  | >20-40 | 43 | 422 | 1.83 (0.94-3.83) |
|  |  | >10-20 | 16 | 197 | 1.54 (0.7-3.51) |
|  |  | ≤10 | 11 | 205 | **1.00 (Ref.)** |
|  | | | | | |
| 2 | Positive | >50 | 55 | 112 | 4.84 (2.76-8.77) |
|  |  | ≤50 | 36 | 90 | 4 (2.2-7.47) |
|  |  |  |  |  |  |
|  | Negative | >90 | 103 | 213 | 4.71 (2.84-8.15) |
|  |  | >80-90 | 58 | 166 | 3.21 (1.86-5.74) |
|  |  | >60-80 | 85 | 420 | 1.88 (1.13-3.25) |
|  |  | >40-60 | 60 | 374 | 1.46 (0.86-2.58) |
|  |  | >20-40 | 81 | 422 | 1.84 (1.11-3.19) |
|  |  | >10-20 | 22 | 197 | 1.14 (0.6-2.18) |
|  |  | ≤10 | 20 | 205 | **1.00 (Ref.)** |
|  | | | | | |
| 3 | Positive | >50 | 54 | 112 | 6.59 (3.62-12.6) |
|  |  | ≤50 | 28 | 90 | 4.01 (2.04-8.1) |
|  |  |  |  |  |  |
|  | Negative | >90 | 80 | 213 | 4.63 (2.64-8.55) |
|  |  | >80-90 | 61 | 166 | 4.18 (2.33-7.85) |
|  |  | >60-80 | 91 | 420 | 2.66 (1.54-4.85) |
|  |  | >40-60 | 78 | 374 | 2.58 (1.49-4.74) |
|  |  | >20-40 | 69 | 422 | 2.19 (1.26-4.02) |
|  |  | >10-20 | 14 | 197 | 0.86 (0.39-1.85) |
|  |  | ≤10 | 18 | 205 | **1.00 (Ref.)** |
|  | | | | | |
| 4 | Positive | >50 | 30 | 112 | 7.51 (3.38-18.51) |
|  |  | ≤50 | 7 | 90 | 2.21 (0.74-6.42) |
|  |  |  |  |  |  |
|  | Negative | >90 | 43 | 213 | 5.14 (2.44-12.21) |
|  |  | >80-90 | 18 | 166 | 2.33 (0.99-5.94) |
|  |  | >60-80 | 35 | 420 | 2.09 (0.98-4.99) |
|  |  | >40-60 | 28 | 374 | 1.94 (0.9-4.69) |
|  |  | >20-40 | 28 | 422 | 1.66 (0.76-4.01) |
|  |  | >10-20 | 10 | 197 | 1.35 (0.52-3.65) |
|  |  | ≤10 | 8 | 205 | **1.00 (Ref.)** |

Abbreviations: CI, confidence interval; FH, family history; GRS, genetic risk score; OR, odds ratio; Ref., reference
^1^ Percentiles of GRS calculated in controls in overall sample
^2^ Model adjusted for sex, age, education, previous colonoscopy, smoking, hormone replacement therapy among women, and body mass index before diagnosis

**Supplementary Figure 1.** Scatter plot and box plot showing the distribution of GRS between different study groups in DACHS


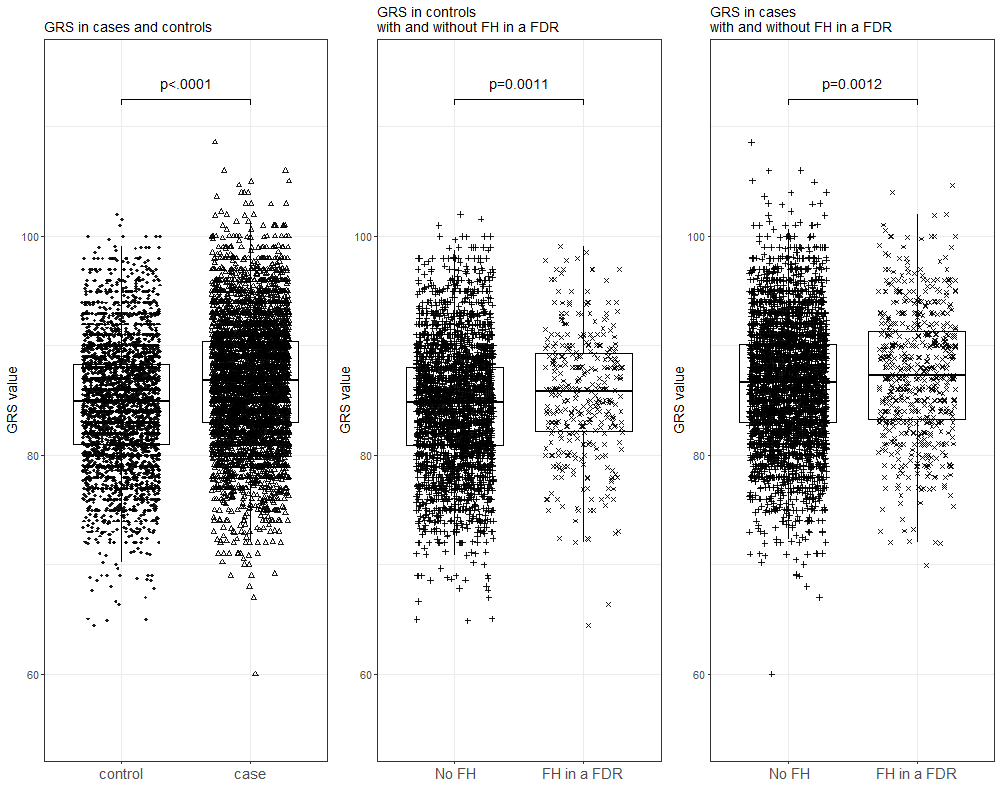


**Supplementary Figure 2.** Scatter plot and box plot showing the distribution of GRS between different study groups in SOCCS


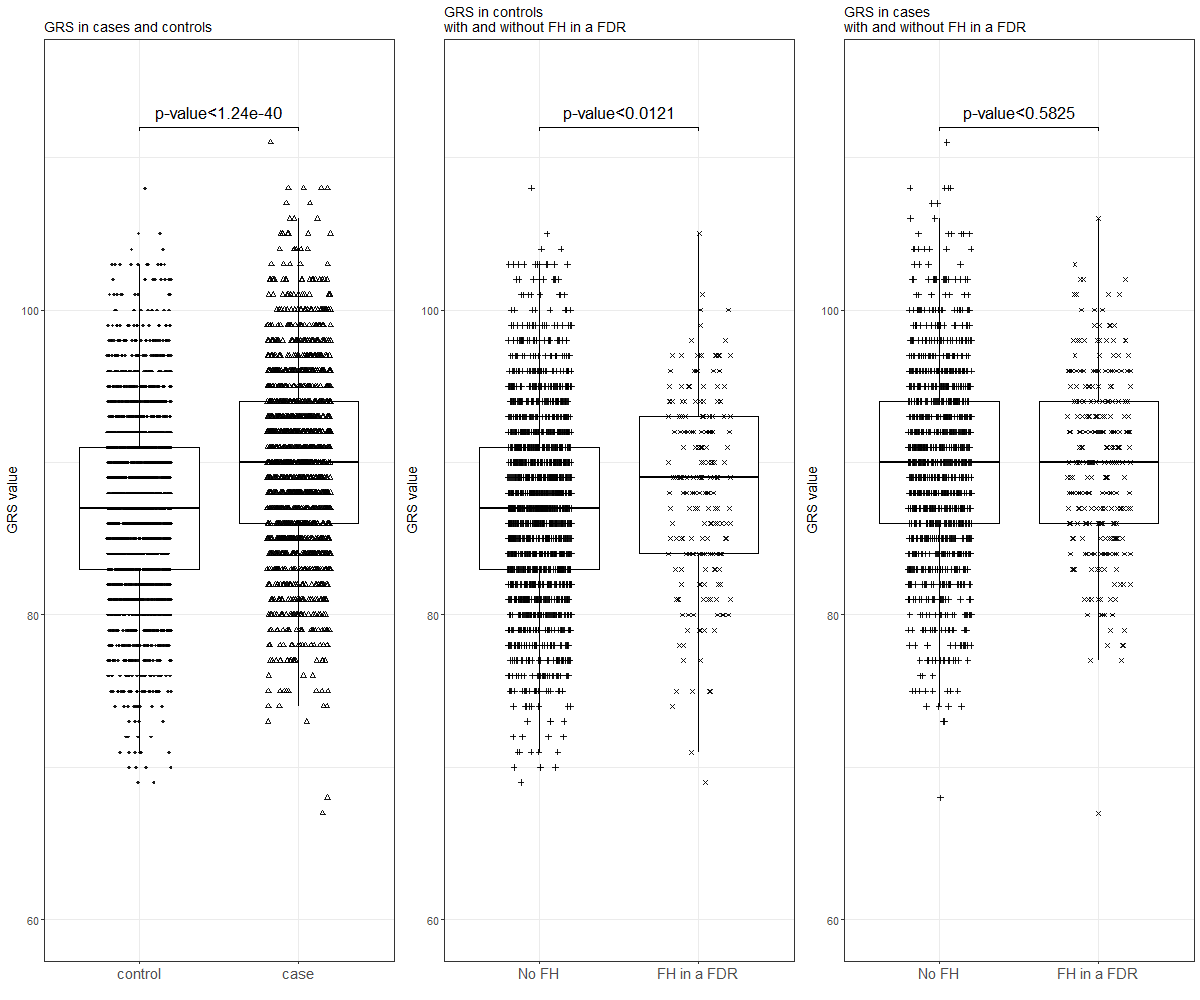


**Supplementary Figure 3.** CRC risk associated with family history (FH) in first-degree relatives (FDR) and genetic risk score (GRS) and affected proportions of population in SOCCS

**Supplementary Figure 3.1.** CRC risk and affected proportion of FH in FDR

**Supplementary Figure 3.2.** CRC risk and affected proportion of dichotomized GRS

**Supplementary Figure 3.3.** CRC risk and affected proportion of a dichotomized GRS and continuous GRS

**Supplementary References**

1. Peters U, Jiao S, Schumacher FR, et al. Identification of Genetic Susceptibility Loci for Colorectal Tumors in a Genome-Wide Meta-analysis. Gastroenterology 2013;144:799-807.e24.

2. Schumacher FR, Schmit SL, Jiao S, et al. Genome-wide association study of colorectal cancer identifies six new susceptibility loci. Nat Commun 2015;6.

3. Weigl K, Chang-Claude J, Knebel P, et al. Strongly enhanced colorectal cancer risk stratification by combining family history and genetic risk score. Clinical Epidemiology 2018;10:9.

4. Huyghe JR, Bien SA, Harrison TA, et al. Discovery of common and rare genetic risk variants for colorectal cancer. Nat Genet 2019;51:76-87.
